# Engineering cell signaling using tunable CRISPR–Cpf1-based transcription factors

**DOI:** 10.1038/s41467-017-02265-x

**Published:** 2017-12-13

**Authors:** Yuchen Liu, Jinghong Han, Zhicong Chen, Hanwei Wu, Hongsong Dong, Guohui Nie

**Affiliations:** 1 Institute of Translational Medicine, Shenzhen Second People’s Hospital, The First Affiliated Hospital of Shenzhen University, Shenzhen, 518039 China; 2 Department of Otolaryngology, Shenzhen Second People’s Hospital, The First Affiliated Hospital of Shenzhen University, Shenzhen, 518039 China

## Abstract

The catalytically dead Cpf1 endonuclease from *Acidaminococcus* sp. *BV3L6* (dAsCpf1) has been used to construct effective transcriptional repressors in bacteria and plants. However, it is still unclear if dAsCpf1 can function in human cells as a transcriptional regulator or a signal conductor. Here, we repurpose the dAsCpf1 system in human cells for a variety of functions, including the activation or repression of gene transcription. Moreover, we construct programmable ligand-controlled dAsCpf1 systems either by coupling crRNAs with engineered riboswitches or by fusing dAsCpf1 proteins with G protein-coupled receptors. These generalizable approaches allow us to regulate the transcription of endogenous genes in response to diverse classes of ligands, thus constructing artificial signaling pathways with rewired cellular input–output behaviors. The systems exhibit signal amplification, an important feature in cell signaling, when multiple crRNAs are processed from a single transcript. The results provide a robust and efficient platform for engineering customized cell signaling circuits.

## Abstract (web summary)

Cpf1 has been repurposed as a transcriptional repressor in bacteria and plants. Here, the authors construct activators and repressors in human cells using Cpf1 coupled to riboswitches and GPCRs.

## Introduction

The CRISPR–Cas9 RNA-guided endonuclease system facilitates rapid and efficient genome editing^1–3^. Using catalytically dead Cas9 (dCas9), this system can be repurposed for the activation or repression of gene transcription in both prokaryotes and eukaryotes^4–7^. In eukaryotic cells, dCas9 can be fused to transcriptional activators or repressors in diverse ways to create unexpected levels of control^8,9^. The use of a repurposed dCas9 system for gene regulation makes it a powerful tool for programming artificial cell signaling networks and pathways^10–13^. Several dCas9-controlled cellular signaling pathways have been engineered that are switchable by external signals, including small molecules^14–16^, light^17–20^, and temperature^20^ changes. Several ongoing studies are evaluating CRISPR as a next-generation platform for gene therapy^21,22^. However, there are still some limitations that need to be addressed, such as high off-target effects^23,24^ and low cellular delivery efficiencies^25^. Moreover, multiple single-guide (sg)RNAs expressed from separate promoters are usually needed to accomplish signal amplification that induces a robust regulation of one targeted gene^5–7,14^. This requires relatively large constructs, which is problematic for current gene therapy.

Improving the CRISPR technique involves exploration of new nucleases with better performances. For example, the recently developed CRISPR–Cpf1 system has overcome some limitations of the CRISPR–Cas9 system. Cpf1 is a smaller endonuclease that can be easily packaged for delivery and can function with a shorter and simpler CRISPR RNA (crRNA) that has low mismatch tolerance^26–29^. It can autonomously process multiple crRNAs from a single transcript to facilitate targeted editing of multiple sequences with T-rich protospacer adjacent motifs (PAMs)^30^. Cpf1 is suitable for targeting AT-rich promoter regions due to its base pairing-dependent PAM recognition^31^. For these reasons, we consider dead Cpf1 (dCpf1) as an attractive tool for genome regulation and signal amplification in cellular engineering. The dCpf1-based transcriptional repressors have already been constructed and tested in bacteria^32,33^ and plants^34^. However, there are only few reports regarding dCpf1s that function in mammalian cells as transcriptional activators or repressors. Rational genetic engineering approaches for controlling dCpf1 systems in an orthogonal and inducible manner are also required. In this study, we repurpose the dCpf1 system in human cells for a variety of biological functions, including the activation or repression of gene expression. More importantly, we also successfully construct programmable ligand-controlled dCpf1 systems and use them to construct new cell signaling pathways with different dynamic ranges.

## Results

### Construction of the codon-optimized dAsCpf1

In previous studies, the Cpf1 proteins from *Acidaminococcus* sp. *BV3L6* (AsCpf1) and from *Lachnospiraceae bacterium ND2006* (LbCpf1) had DNA cleavage activity in human cells^29,30^. Although AsCpf1 was less effective than LbCpf1 in DNA cleavage, it bound DNA more tightly than did LbCpf1^34^. We reasoned, therefore, that dAsCpf1 may be more suitable for regulating gene transcription. The dAsCpf1 (D908A) was codon optimized (Supplementary Note 1), fused to three copies of a nuclear localization sequence, and expressed under the control of the human elongation factor 1 A-1 (hEF1A-1) promoter. The crRNA was expressed from the RNA polymerase III U6 promoter. The plasmid maps were shown in Supplementary Fig. 1a–e.

### Construction of the dCpf1-based transcriptional repressors

**Fig. 1:**
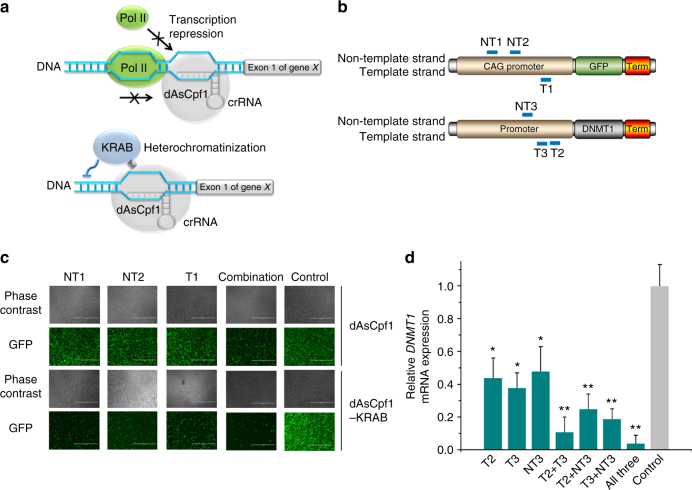
The dAsCpf1-based transcriptional repressors effectively silenced transcription. **a** Design of the dAsCpf1-based transcriptional repressor. dAsCpf1 could specifically interfere with RNA polymerase binding and scanning processes. To generate a fusion protein capable of mediating stronger transcriptional repression, we directly tethered the KRAB repression domain to the C terminus of dAsCpf1. The fusion protein bound to the crRNA and formed a transcriptional repression complex. **b** Binding regions between the designed crRNAs and their DNA targets. Base pairing nucleotides of the crRNAs that bound to either the template DNA strand or the non-template DNA strand are shown in blue. **c** The dAsCpf1-based transcriptional repressors displayed RNA-guided transcriptional repression as detected by fluorescent microscopy. Representative images of the transfected cells are shown. Scale bar, 1000 μm. **d** For *DNMT1*, we designed three crRNAs and crRNA arrays expressing all the possible pairs of these crRNAs, and assayed transcriptional repression by qRT-PCR. Reported data are the mean ± SD from five separate experiments. *P < 0.05 or **P < 0.01 compared to the non-target crRNA controls, by paired, one-sided *t*-tests

Because dCas9–sgRNA effectively blocked transcription, we first tested if we could knock down targeted gene expression by coexpression of a crRNA and dAsCpf1 (Fig. 1a). A green fluorescent protein gene (*GFP*) expression cassette driven by a CAG promoter was constructed and inserted into the genome of the human embryonic kidney 293T cell line (HEK-293T). Three crRNAs (Supplementary Table 1) complementary to different regions of the CMV early enhancer chicken beta actin (CAG) promoter sequence (Fig. 1b) were designed to either bind to the template DNA strand or to the non-template DNA strand. Because the Cpf1 can process multiple crRNAs within a single transcript^30,33^, we also encoded a combination of these three crRNAs under the control of a single promoter in one transcript (Supplementary Fig. 2). After 48 h of transient transfection of the related plasmids into HEK293T cells stably expressing *GFP*, the regulatory efficiency was investigated by observing and quantificating *GFP* expression (Supplementary Fig. 3a–c). Surprisingly, the three crRNAs had little effect on *GFP* expression compared to the non-target crRNA control (Fig. 1c; Supplementary Fig. 3a). However, modest transcriptional repression in the presence of the crRNA array expressing all three crRNAs was observed (Fig. 1c; Supplementary Fig. 3a), indicating that synergistic effects were induced by binding of multiple crRNA–dAsCpf1 complexes to a single promoter. We confirmed that dAsCpf1 was a highly specific regulator, similar to the wild-type AsCpf1, because the *GFP* messenger RNA (mRNA) was the only transcript that was significantly inhibited by the *GFP*-targeting crRNA array (Supplementary Fig. 4). To optimize the dAsCpf1-based repressors for the induction of robust transcriptional repression, the dAsCpf1 was fused with the Krüppel-associated box (KRAB) domain (Fig. 1a; Supplementary Fig. 1b). This design was suggested by the observation that fusion of dCas9 to KRAB remarkably enhanced the repression efficiency^35^. The expression of each of the three crRNAs alone induced decreases in *GFP* expression when coexpressed with dAsCpf1–KRAB in HEK293T cells (Fig. 1c; Supplementary Fig. 3b). This observation suggested that the activity of dAsCpf1-based repressors could be improved if a strong transcriptional repressor domain was used. In addition, in experiments with the crRNA array expressing a combination of all the crRNAs, inactivation of *GFP* was also enhanced (Fig. 1c; Supplementary Fig. 3b). To determine whether endogenous genes could be repressed by the dAsCpf1–KRAB system, three target sequences were designed for the promoter region of the DNA (cytosine-5)-methyltransferase 1 (*DNMT1*) (Fig. 1b; Supplementary Table 1). About 48 h after transient transfection of the related plasmids into HEK293T cells, repression of *DNMT1* was observed, as measured by qRT-PCR, and stronger repression was achieved when a combination of these crRNAs was expressed (Fig. 1d). Coexpression of two repressor crRNAs resulted in synergistic repression of the *DNMT1* expression and slightly more inhibition was achieved by using all the crRNAs (Fig. 1d). In contrast, no obvious effect was observed with any of the tested crRNAs when dAsCpf1, without the KRAB domain, was targeted to *DNMT1*. The crRNA array expressing all three crRNAs has similarly neutral effect on *DNMT1* repression (Supplementary Fig. 5a). These results demonstrated that dAsCpf1-based transcriptional repressors efficiently repressed the transcription of target genes, and that robust transcriptional repression could be achieved through the use of multiple synergistic crRNAs.

### Construction of the dCpf1-based transcriptional activators

**Fig. 2:**
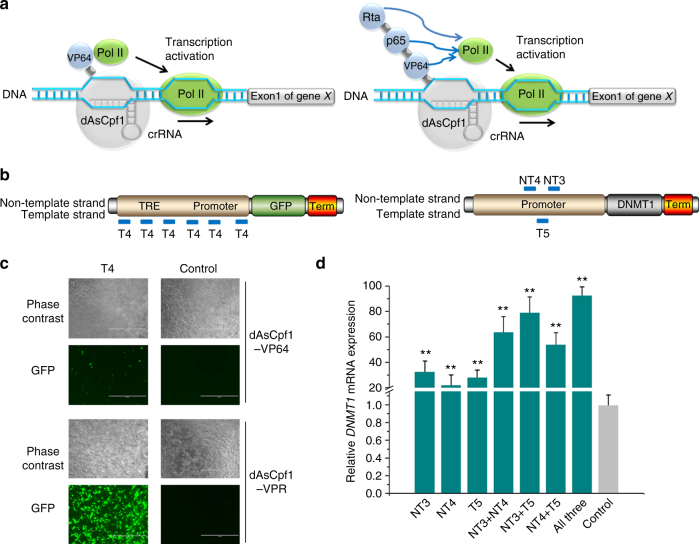
The dAsCpf1-based transcriptional activators effectively increased transcription. **a** Design of the dAsCpf1-based transcriptional activator. To generate a fusion protein capable of transcriptional activation, we directly tethered the tripartite VP64 to the C terminus of dAsCpf1. To generate a fusion protein capable of mediating stronger transcriptional activation, we also tethered the tripartite VPR activator to the C terminus of dAsCpf1. VPR is a fusion of VP64, p65, and RTA. The fusion protein was bound to the crRNA and formed a transcriptional activation complex. **b** Locations of crRNAs targeted to the gene promoter. Blue lines indicate base pairing nucleotides of the crRNAs that bound to either the template DNA strand or the non-template DNA strand. **c** The dAsCpf1-based transcriptional activators displayed RNA-guided transcriptional activation as detected by fluorescent microscopy. Representative images of the transfected cells are shown. Scale bar, 1000 μm. **d** For *DNMT1*, we designed three independent crRNAs and crRNA arrays expressing all the possible pairs of these crRNAs, and assayed transcriptional activation by qRT-PCR. Reported data are the mean ± SD from five independent experiments. ^**^ *P* < 0.01 compared to the non-target crRNA control, by paired, one-sided *t*-tests

We determined if dAsCpf1-based transcriptional activators could upregulate gene expression in HEK293T cells using single or multiple crRNAs (Fig. 2a). A stable reporter HEK293T cell line containing a tetracycline responsive element (TRE) promoter-driven *GFP* expression cassette was produced. Because the basal expression level of the TRE promoter was extremely low, it was easy to detect significant activation from this promoter. To promote transcriptional activation, we fused four tandem copies of herpes simplex viral protein 16 (VP64, a widely used domain in dCas9-based activation systems^5–7^) to the codon-optimized dAsCpf1 (Supplementary Fig. 1c). We designed one crRNA that bound to the repetitive DNA sequences (six copies) within the TRE promoter (Fig. 2b; Supplementary Table 1) and tested the capacity of the dAsCpf1–VP64 fusion to activate expression of *GFP* at 48 h after transient transfection. To our surprise, only minimal activation was observed with the tested crRNA (Fig. 2c; Supplementary Fig. 3c), which was inconsistent with previous findings that gene activation with the dCas9–VP64 had relatively large effects^5–7,14^. We suggest that dAsCpf1–VP64 may be a weak transcriptional activator. Therefore, to construct a robust gene activation system, dAsCpf1 was fused at the C terminus with the tripartite VPR activator^36^, a fusion construct of the VP64, p65, and Epstein–Barr virus R transactivator (RTA) domains (Fig. 2a; Supplementary Fig. 1d). As expected, the crRNA induced increases in *GFP* expression (Fig. 2c; Supplementary Fig. 3c). To determine whether endogenous genes could be activated by the dAsCpf1–VPR system, we designed three crRNA sequences for the *DNMT1* gene (Supplementary Table 1) and constructed crRNA arrays expressing a combination of these crRNAs using one promoter. These crRNAs bound to sequences near the transcription start site of the *DNMT1* promoter (Fig. 2b). At 48 h after transient transfection, the expression of each crRNA resulted in strong activation of *DNMT1* expression compared to the non-target crRNA control (Fig. 2d). Similarly, coexpression of dAsCpf1–VPR with three crRNAs (NT3, NT4, and T5) as well as with subsets of two of these three crRNAs enabled synergistic activation of DNMT1 transcripts (Fig. 2d). On the other hand, no significant effect was observed with any of the tested crRNAs (or the crRNA array) when dAsCpf1, without the VPR activator, was targeted to *DNMT1* (Supplementary Fig. 5b). These results demonstrated that dAsCpf1-based transcriptional activators efficiently activated transcription of target genes, and that robust transcriptional activation can be achieved through the use of multiple synergistic crRNAs.

To further confirm that the above reported gene regulatory effects were independent of specific cell lines or specific gene loci, transcriptional activation (Supplementary Fig. 6a) and repression (Supplementary Fig. 6b) of the human vascular endothelial growth factor A (*VEGFA*) gene in HeLa cells were performed using crRNA–dAsCpf1–VPR and crRNA–dAsCpf1–KRAB. Both the transcriptional activators and repressors were observed to be effective, thus indicating the versatility of the dAsCpf1-based factors.

### Engineering cell signaling using the crRNA-riboswitch

**Fig. 3:**
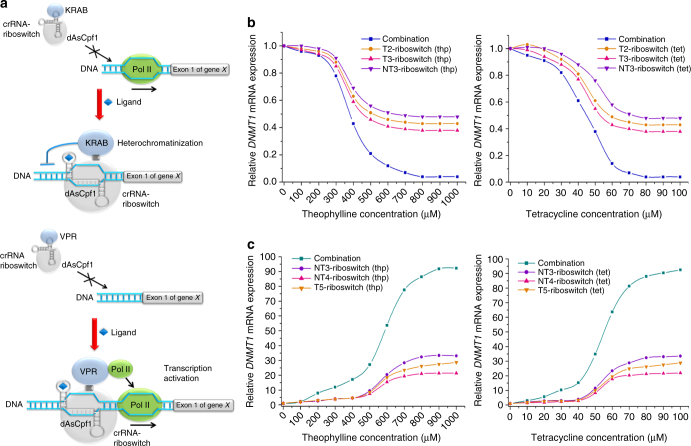
Design and characterization of the riboswitch-controlled dAsCpf1-based transcription factors. **a** General illustration of the mechanisms by which the redesigned switchable crRNAs regulate gene transcription in vivo. After transcription, the guide region of crRNA is paired within the antisense stem and the crRNA is in the “off” state. In the presence of ligand, the conformation of the redesigned crRNA is switched to the “on” state, and the guide region of the crRNA binds to its target DNA and thus turns the transcription of targeted gene off and on through the dAsCpf1–KRAB protein and the dAsCpf1–VPR protein, respectively. **b**, **c** The relative expression levels of *DNMT1* mRNA were detected using real-time qPCR in HEK293T cells that respond to theophylline or tetracycline across different concentrations. Reported values are the averages of five independent experiments

Although the dAsCpf1-based transcription factors described above provided tools for efficient gene regulation, there was still the need to couple these artificial transcription regulators with signal-responsive modules that directly detected small molecules. To apply dAsCpf1 tools for engineering cellular signaling circuits, riboswitches^14,37^ that recognize specific stimuli have been inserted into crRNAs to alter gene expression in a controllable way. The redesigned crRNA uses the traditional module to recruit the dAsCpf1-based regulator and the riboswitch-based biosensor to recognize a specific ligand. In the absence of the specific ligand, the guide region of the crRNA pairs with the antisense stem and therefore cannot bind to its target DNA. Upon sensing the specific ligand by the RNA riboswitch, a conformational change allows the guide region of the crRNA to bring the dAsCpf1-based regulator to the target gene region, thereby regulating gene transcription (Fig. 3a; Supplementary Fig. 7a, b). We incorporated a theophylline aptamer^38^ into the 3′ end of each of the crRNAs targeting *DNMT1* (Supplementary Table 1) and tested if the addition of theophylline affected the transcription of *DNMT1* in HEK293T cells after transient transfection with the related plasmids. Each reprogrammed crRNA–dAsCpf1–KRAB complex showed an efficient silencing effect in the presence of theophylline at 48 h after transient transfection. A dose-dependent repressive effect upon treatment with theophylline was observed (Fig. 3b). As a result of the synergy achieved among all three of the individually utilized crRNA–dAsCpf1–KRAB complexes, we observed a larger and quicker decrease in the construct expressing the crRNA-riboswitch array. All the reprogrammed crRNAs induced significant increases in *DNMT1* mRNA expression when combined with dAsCpf1–VPR and a dose-dependent effect was also observed for each crRNA-riboswitch or the crRNA-riboswitch array (Fig. 3c). The highest dynamic range of the *DNMT1* gene was achieved with the crRNA-riboswitch array.

To test if this could be reversed, we also performed time course measurements of dAsCpf1-mediated regulation of *DNMT1* after washing the ligands away. After a delay of 3 h, the changing trends of *DNMT* mRNA reversed. After 15 h, *DNMT1* mRNA levels in all groups were uniformly reversed to the same level as the original state (Supplementary Fig. 8a, b).

The modularity of the crRNA-riboswitch design was tested by replacing the theophylline aptamer with a tetracycline aptamer^38^ (Supplementary Table 1), leaving a stem sequence identical to the previous design. Similar repression (Fig. 3b) and activation (Fig. 3c) effects were observed when each crRNA-riboswitch or the crRNA-riboswitch array was coexpressed with the dAsCpf1–KRAB and dAsCpf1–VPR, respectively. These crRNA-riboswitch systems exhibited little basal activity without the ligands (Supplementary Fig. 9a, b). In addition, the systems expressing the control crRNA-riboswitches did not have any ligand-responsive characteristics (Supplementary Fig. 10a–d). These data demonstrated that the crRNA-riboswitch–dAsCpf1 complex regulated the transcription of endogenous genes in response to external riboswitch-responsive signals and thereby altered cellular signaling.

### Engineering cell signaling with dAsCpf1-based receptors

**Fig. 4:**
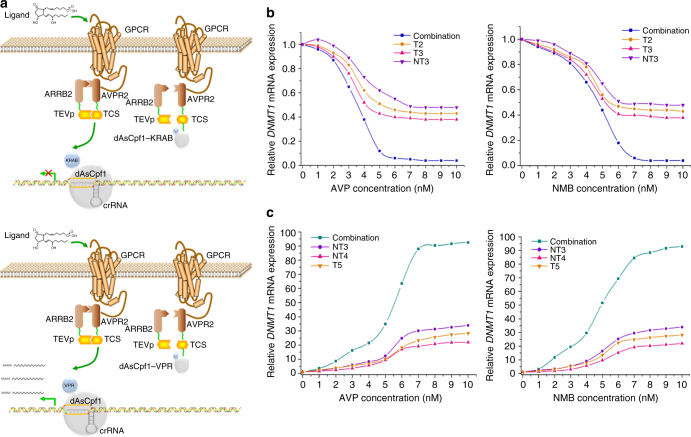
Design and characterization of the GPCR-controlled dAsCpf1-based transcription factors. **a** The design schemes for coupling dAsCpf1 function to the activity of GPCRs. We fused the dAsCpf1–KRAB or dAsCpf1–VPR to the C terminus of GPCR. An adaptor protein, ARRB2, was fused to the TEVp protease. Ligand binding to the GPCR stimulated recruitment of the ARRB2–TEVp fusion construct and cleavage at the TCS sequence, triggering the release of the tethered transcription factors, dAsCpf1–KRAB or dAsCpf1–VPR. The dAsCpf1-based transcription factor then entered the nucleus and regulated endogenous gene transcription. **b**, **c** The relative expression levels of *DNMT1* mRNA were detected using real-time qPCR in HEK293T cells that respond to AVP or NMB across different concentrations. Reported values are the averages of five independent experiments. GPCR G protein-coupled receptor

While the above data demonstrated the feasibility of the riboswitch mechanism in controlling dAsCpf1 activity, ligand recognition was limited to detection of a small number of molecules which have available RNA aptamers. To create an alternative approach that differed in extracellular signal sensing ability, we repurposed the G protein-coupled receptor (GPCR)^39^ as a biosensor for converting the input signals into gene expression responses using dAsCpf1-based transcription factors (Fig. 4a). Based on previous studies that monitored the activation of GPCRs^40–42^, we used the Tango design for proteolytic coupling of dAsCpf1 function to GPCR–β-arrestin interactions. A fusion protein consisting of the human arginine vasopressin receptor 2 (AVPR2) joined at its C terminus to a dAsCpf1-based transcription factor was synthesized. An adaptor protein, human β-arrestin2 (ARRB2), which interacts with AVPR2 upon ligand activation, was fused to the N1a protease from tobacco etch virus (TEVp). Moreover, a specific seven amino-acid cleavage site for the TEVp (TCS) was introduced between the AVPR2 and the dAsCpf1 effector. The ligand-dependent recruitment of ARRB2–TEVp to the AVPR2–dAsCpf1 and the subsequent proteolytic cleavage allowed the dAsCpf1 effector to enter the nucleus and regulate the endogenous targeted genes (Fig. 4a). HEK293T cells were transiently transfected with the plasmids expressing the fusion proteins based on AVPR2–dAsCpf1–KRAB. Western blot analysis of subcellular fractions revealed that this fusion protein was primarily localized in the cytoplasm. In the cells treated with 5 or 10 nM arginine vasopressin (AVP, the AVPR2 agonist), the protein was translocated to the nucleus (Supplementary Fig. 11a). The crRNAs targeting *DNMT1* were used in this study, and the qRT-PCR results showed a decrease in *DNMT1* mRNA expression in the presence of AVP at 48 h after transient transfection (Fig. 4b). In response to AVP, *DNMT1* mRNA exhibited a dose–response profile, suggesting that the constructed system could be used to generate dynamic cell responses to environmental stimuli (Fig. 4b). In addition, the expression of dAsCpf1–KRAB with a single crRNA array led to an accelerated reduction in *DNMT1* expression (Fig. 4b). These results demonstrated that this approach could be used to build artificial cellular signaling response systems with tunable strengths by using engineered crRNA expression cassettes.

We also measured how an engineered GPCR based on dAsCpf1–VPR affects gene activation by transfecting with related plasmids. Alterations in the localization of dAsCpf1–VPR were observed with AVP treatment (Supplementary Fig. 11b). AVP activated *DNMT1* mRNA expression at 48 h after transient transfection in a dose–response manner, a feature that is critical for achieving efficient GPCR-mediated activations (Fig. 4c). Multiple crRNAs generated by the dAsCpf1–VPR also acted synergistically to stimulate robust transcriptional induction of *DNMT1* expression (Fig. 4c).

To further demonstrate the modularity of the dAsCpf1–Tango design platform, we used neuromedin B receptor (NMBR), another GPCR that senses NMB. The C terminus of NMBR was fused with a tail sequence from AVPR2. Like the AVPR2 Tango, the NMBR Tango exhibited NMB-responsive *DNMT1* repression (Fig. 4b) or activation at 48 h after transient transfection (Fig. 4c), suggesting that the Tango design based on the GPCR–dAsCpf1 was also modular. These dAsCpf1–GPCR systems exhibited little basal activity without the addition of ligands (Supplementary Fig. 12a, b). Moreover, GPCR systems expressing the control crRNA did not display any ligand-responsive behaviors (Supplementary Fig. 13a–d). Because GPCRs can detect diverse ligands, this approach expands the detection range of dAsCpf1-based networks to a large number of biological signals.

In addition, we also investigated whether the most widely used system CRISPR–dCas9 could be harnessed to regulate transcription from GPCRs. To accomplish this objective, GPCR–TCS–dAsCpf1–KRAB and GPCR–TCS–dAsCpf1–VPR were replaced with GPCR–TCS–dCas9–KRAB and GPCR–TCS–dCas9–VPR, respectively. We also designed several sgRNAs to inactivate or activate *DNMT1* expression (Supplementary Table 2). However, the dCas9–GPCR design displayed very high leakiness with significant *DNMT1* regulation without ligand treatment (Supplementary Fig. 14a, b).

## Discussion

Targeted and inducible regulation of endogenous gene expression is a powerful method for interrogating gene function and rewiring signaling networks. Adding the dCas9-based regulator to a promoter or coding region of a gene activated or inactivated its transcription in both bacteria and mammalian cells^4–9^. However, the size of the dCas9 transgene system is large^25^, thus limiting the utility of the system as a tool for reprogramming cellular information. In contrast, the expression cassette(s) of the dCpf1 system are much simpler than that of the dCas9 system^26–34^. We hypothesized that a smaller system may make processing simpler and more exact.

In this study, our data provided proof of principle that dAsCpf1 fused to transcriptional effector domains regulated gene transcription in a similar way to that of dCas9 in human cells. During the course of the peer review of this manuscript, another paper describing CRISPR–dLbCpf1-based transcription factors was published online^43^. In the study, drug-inducible dLbCpf1-based activators were used to simultaneously upregulate the transcription of multiple genes.

External ligands not associated with specific gene regulation can be redirected to control targeted gene expression patterns through the use of inducible dAsCpf1-based transcription factors. One obvious advantage is that these systems can amplify the input signals by cleaving and releasing multiple crRNAs from a single transcript. These efforts facilitated the construction of a compact and effective CRISPR–Cas system for in vivo applications of cellular engineering. Rewiring human cellular input–output signals using these modular systems would increase our understanding of how cellular networks function to make decisions and how they can be rewired. The two generalizable approaches we provided for fine-tuning dAsCpf1 effector activity may enable cell-based therapies for treating diverse diseases.

The riboswitch experiments showed similar small molecule binding responses (concentration ranges) and regulatory activities between the crRNA-riboswitches–dCpf1 and the previously reported sgRNA-riboswitches–dCas9^14^. The comparable binding properties were expected, as they used the same RNA aptamers. In the GPCR experiments, dCas9 displayed much higher leakiness than dCpf1. This could be explained by the speculation that fusing a large domain such as dCas9 to a GPCR affected the receptor conformation. The advantages and disadvantages between the two systems will be further compared in future studies.

## Methods

### Plasmid construction

DNA encoding the human sequence-optimized dAsCpf1 nuclease harboring the inactivating D908A substitution was chemically synthesized and cloned into a plasmid containing a HEf1A promoter to yield plasmid dAsCpf1(D908A)–crRNA. The dAsCpf1 sequence fused with the KRAB domain sequence was then inserted into the same backbone to form plasmid dAsCpf1(D908A)–krab–crRNA. The dAsCpf1(D908A)–VP64–crRNA and dAsCpf1(D908A)–VPR–crRNA constructs were assembled by fusing dAsCpf1(D908A) with VP64 and VPR, respectively, at the C terminus. The plasmid maps are shown in Supplementary Fig. 1a–d. The original crRNAs were designed using the online design tool “Cas-Designer” (http://www.rgenome.net/cas-designer/). The designed complimentary DNA (cDNA) sequence for each crRNA was synthesized and inserted into the corresponding plasmid expressing both dAsCpf1 and crRNA. The RNA secondary structures of the designed crRNA-riboswitchs are shown in Supplementary Fig. 7a, b. AVPR2–TCS–dAsCpf1–VPR and AVPR2–TCS–dAsCpf1–KRAB were assembled by fusing AVPR2 (GenBank accession number NM_000054) with dAsCpf1–VPR and dAsCpf1–KRAB, respectively. The TCS sequence ENLYFQS was inserted between AVPR2 and dAsCpf1 and was flanked with GS linkers. The coding region of ARRB2 (GenBank accession number NM_004313) was fused with a DNA fragment encoding the catalytic domain of the TEV protease (GenBank accession number M15239). ARRB2 was flanked by two nuclear export signals (NES: LALKLAGLDI) to ensure cytoplasmic localization of the fusion protein. NMBR (Addgene #66445; Addgene, Cambridge, MA, USA) was chemically synthesized and cloned into a pcDNA3.0 vector. The V2 sequence derived from AVPR2 was inserted between NMBR and dAsCpf1–KRAB/dAsCpf1–VPR as a primer overhang by InFusion (Clontech, Mountain View, CA, USA) cloning. The human codon-optimized *Streptococcus pyogenes* dCas9 sequence was derived from pcDNA–dCas9–HA (plasmid #61355; Addgene) and vectors expressing dCas9–GPCRs were constructed using similar methods as described above.

### Cell culture and cell transfection

HEK293T and HeLa cells were purchased from American Type Culture Collection (ATCC) and maintained in DMEM medium supplemented with 10% fetal bovine serum (Invitrogen) in the presence of 5% CO_2_ at 37 °C in an incubator. The HEK293T pCAG–GFP cell line and the HEK293T pTRE-GFP cell line were obtained by transfecting cells with the corresponding plasmids and selecting positive clones with G418. For transient transfection experiments, 200,000 cells were seeded in 12-well plates the day before transfection, and cells were treated with the mixtures of plasmids (1 µg/μL) using Lipofectamine 2000 Transfection Reagent (Invitrogen) according to the manufacturer’s protocols, after they reached 70–80% confluency. For inducible expressions, test ligands in growth media were added 6 h post transfection.

### RNA extraction and real-time quantitative PCR

About 48 h post transfection, TRIzol reagent (Invitrogen) was used to extract total RNA from cells transfected with the plasmids according to the manufacturer’s protocols. The cDNA was synthesized from total RNA with the RevertAid First Strand cDNA Synthesis Kit (Fermentas, Hanover, MD, USA). The real-time quantitative PCR reactions were performed on an ABI PRISM 7000 Fluorescent Quantitative PCR System (Applied Biosystems, Foster City, CA, USA) using the All-in-One qPCR Mix (GeneCopoiea Inc, Rockville, MD, USA). The PCR cycling parameters were as follows: 95 °C for 15 min, followed by 40 cycles of 94 °C for 15 s, 55 °C for 30 s, and 72 °C for 30 s. The primer sequences were as follows: DNMT1 primers, forward, 5′-GAGGAGGGCTACCTGGCTAA-3′, and reverse, 5′-GCTTAGCCTCTCCATCGGAC-3′; GFP primers, forward, 5′-ACGTAAACGGCCACAAGTTC-3′, and reverse, 5′-AAGTCGTGCTGCTTCATGTG-3′; and TBP primers, forward, 5′-CCCGAAACGCCGAATATAATCC-3′, and reverse, 5′-AATCAGTGCCGTGGTTCGTG-3′. Each experiment was performed five times. TBP was used as the internal control and the data were normalized to the expression of TBP. Relative gene expression was calculated using the Delta-Delta-Ct (^ΔΔ^Ct) algorithm.

### In vitro detection of GFP expression

The cells were cultured with normal growth medium, transfected with the plasmids, and then examined for GFP expression after 48 h using fluorescent microscopy (MicroPublisher 3.3 RTV; Olympus, Tokyo, Japan). Images were captured in auto-exposure mode. For fluorescence measurements, the cells were trypsinized to a single-cell suspension and gated with a mKate-only-positive population (top 80% used for transient transfection experiments). GFP expression intensity was then measured.

### Analysis of the specificity of dAsCpf1

Total RNA was extracted and mixed with oligo (dT)^25^ Dynabeads, and then mRNA was purified based on the manufacturers’ procedure (Invitrogen). The purified mRNA was first fragmented using divalent cations before being employed for library preparation. A linker was ligated to the fragmented RNA using truncated T4 RNA ligase 2 (NEB) according to the manufacturer’s protocols, and the RNA was then reverse transcribed to DNA using SuperScript III (Thermo Scientific, Scotts Valley, CA, USA) and circularized using Circligase (EpiBio/Illumina Madison, WI, USA). Barcodes were added by PCR with Phusion polymerase (Thermo Scientific). The DNA library was sequenced on the Illumina HiSeq 2500 (Illumina). Reads were processed using the Cufflinks v2.1.1, and fold-changes were also calculated based on the fragments per kilobase of transcript per million mapped reads (FPKM) values.

### Western blot analysis

Cells were washed in PBS and lysed in RIPA buffer (50 mM Tris-HCl pH 7.2, 150 mM NaCl, 1% NP40, 0.1% SDS, 0.5% DOC, 1 mM PMSF, 25 mM MgCl_2_, and supplemented with a phosphatase inhibitor cocktail). Cells were harvested and membrane and nuclear protein fractions were separated using the Subcellular Protein Fractionation Kit for Cultured Cells (Pierce, Life Technologies) according to the supplier’s protocols. The protein concentration was determined using the BCA protein assay. Equal amounts of whole protein extract were electrophoresed onto SDS–polyacrylamide gels and then transferred to PVDF membranes (Millipore, Billerica, MA). Samples were blocked in 5% dry milk and incubated overnight with the primary antibodies against AsCpf1 (1:500; Sigma-Aldrich SAB4200756, St. Louis, MO, USA), Pan-Cadherin (1:1000; Abcam ab6528, Cambridge, MA, USA) and HDAC1 (1:1000; Sigma-Aldrich H6287, St. Louis, MO, USA). Then, the samples were incubated with horseradish peroxidase-conjugated secondary antibody (Amersham, Piscataway, NJ) and immunoblots were developed with Super Signal chemiluminescence reagents (Pierce Chemical Co.).

### Statistical analyses

Statistical analyses were conducted using the Student’s *t*-test and *P* < 0.05 was considered statistically significant. All statistical tests were performed using SPSS, version 17.0 software (SPSS, Chicago, IL, USA).

### Data availability

All relevant data are available from the corresponding author on reasonable request. Plasmid sequences are listed in Supplementary Tables and Supplementary Note 1.

## Electronic supplementary material

Supplementary Information
