# Supplementary material for: Engineering cell signaling using tunable CRISPR–Cpf1-based transcription factors
Source: Nat Commun. 2017 Dec 13;8:2095. doi: 10.1038/s41467-017-02265-x (PMC5727435; doi:10.1038/s41467-017-02265-x)
Supplement: Supplementary file 1 — Supplementary Information [file 41467_2017_2265_MOESM1_ESM.pdf]

a

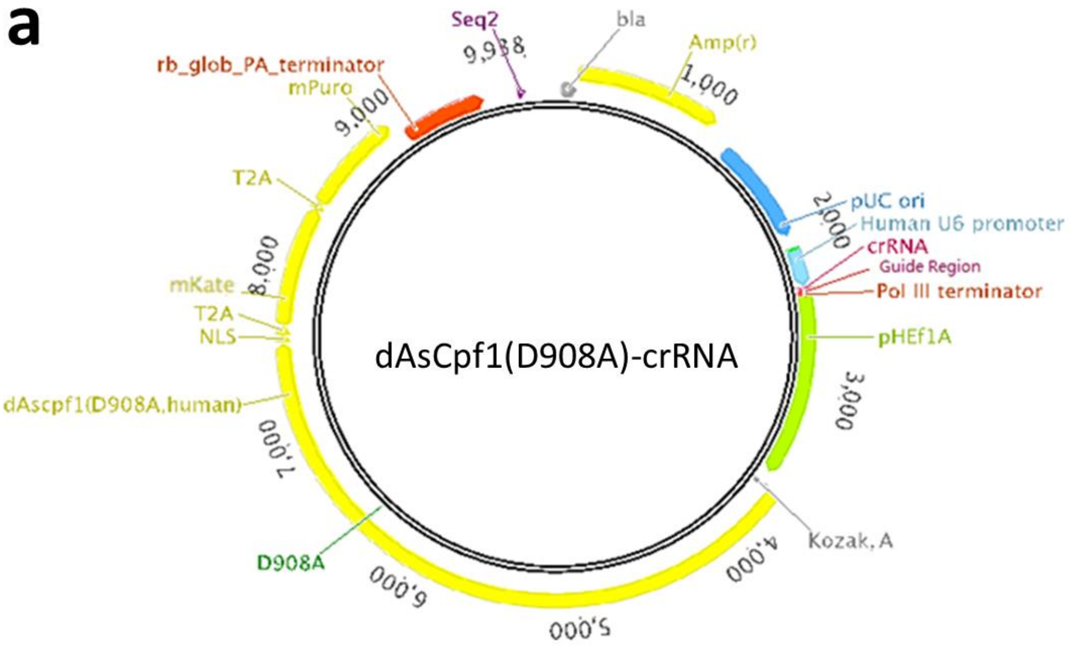
Seq2
9,938
bla
Amp(r)
1,000
rb_glob_PA_terminator
mPuro
9,000
T2A
pUC ori
2,000
Human U6 promoter
crRNA
Guide Region
Pol III terminator
8,000
mKate
T2A
NLS
pHEf1A
dAsCpf1(D908A)-crRNA
dAscpf1(D908A,human)
3,000
7,000
Kozak, A
D908A
4,000
6,000
5,000

b

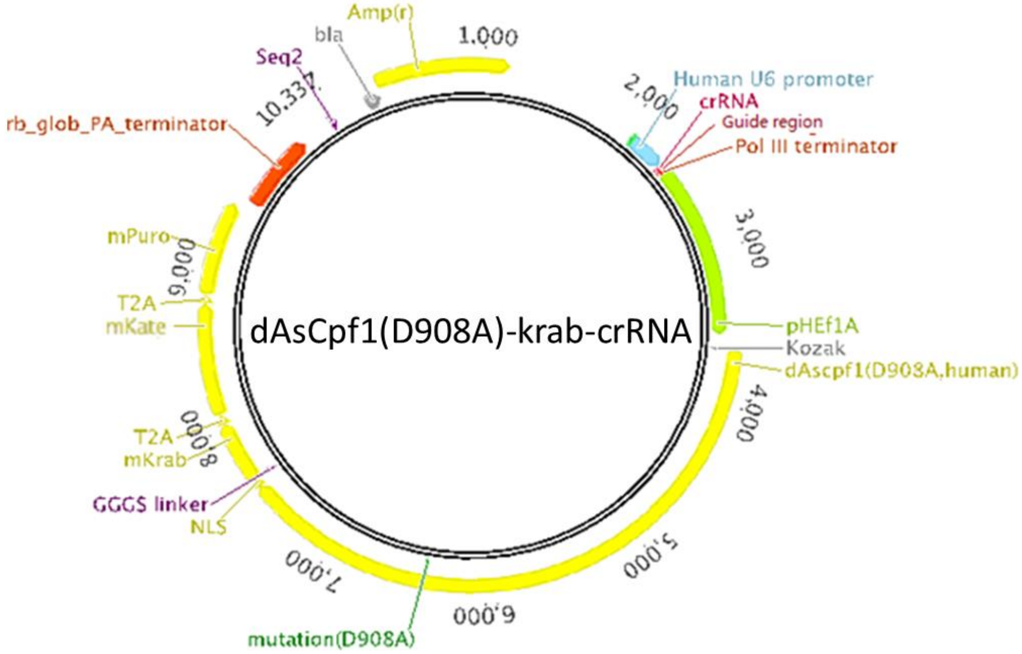
Amp(r)
1,000
bla
Seq2
Human U6 promoter
2,000
crRNA
Guide region
Pol III terminator
rb_glob_PA_terminator
3,000
mPuro
9,000
T2A
mKate
dAsCpf1(D908A)-krab-crRNA
pHEf1A
Kozak
dAscpf1(D908A,human)
4,000
8,000
T2A
mKrab
GGGS linker
NLS
5,000
7,000
6,000
mutation(D908A)

c

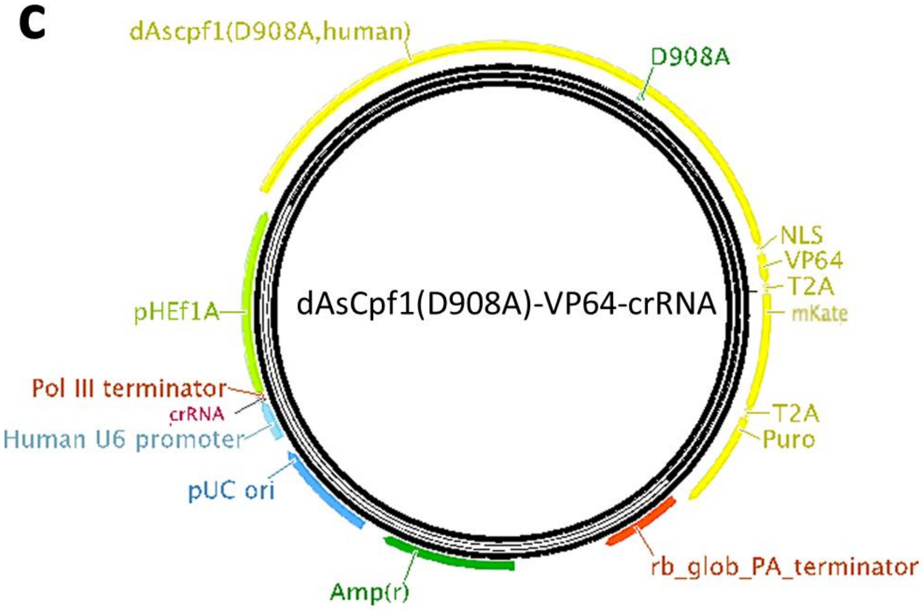

d

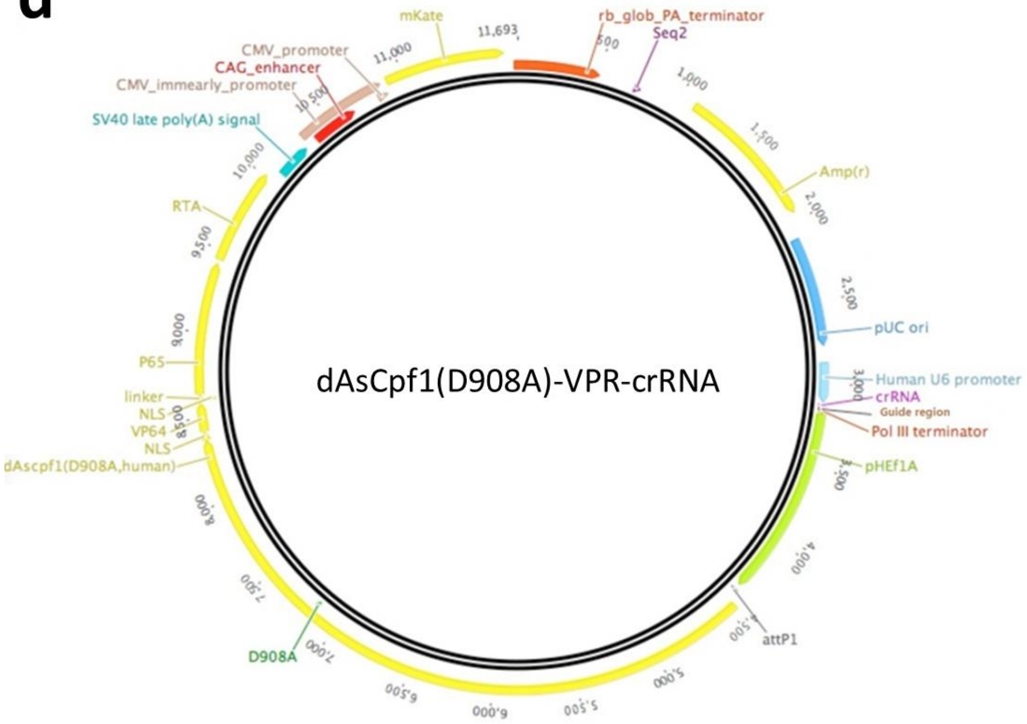

Supplementary Figure 1. Set of plasmids used for expressing dAsCpf1-based transcription factors. Various modules such as promoters, gene coding regions, mutation sites and resistance markers were shown in these plasmid maps. a) dAsCpf1(D908A)-crRNA expression vector. b) dAsCpf1(D908A)-krab-crRNA expression vector. c) dAsCpf1(D908A)-VP64-crRNA expression vector. d) dAsCpf1(D908A)-VPR-crRNA expression vector.

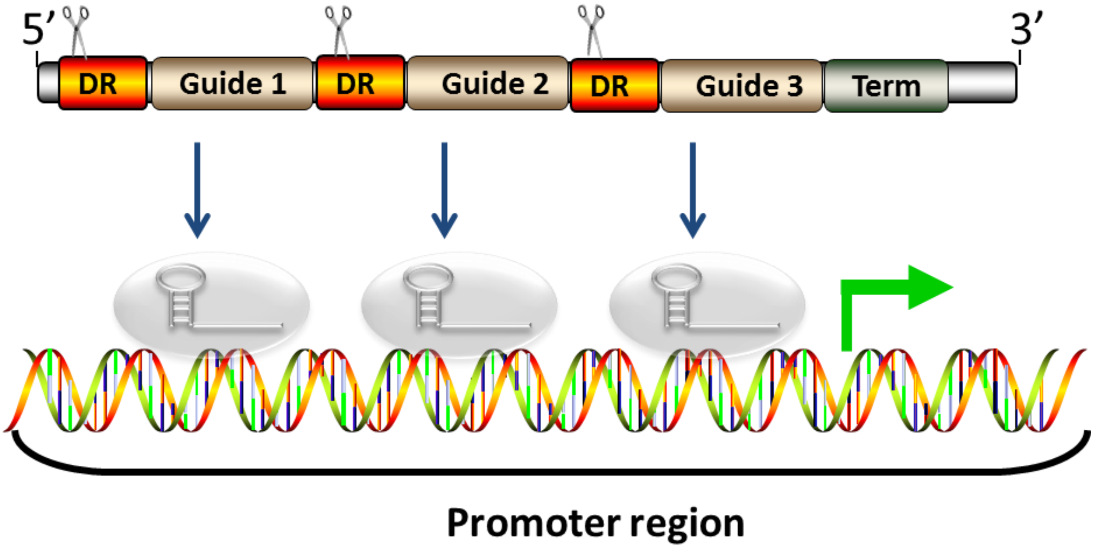

Supplementary Figure 2. Schematic of crRNA-array processing for dCpf1. When the crRNA array was coexpressed with dCpf1, all three crRNAs were released from one single transcript. Cleavage sites indicated with scissors. DR, direct repeat.

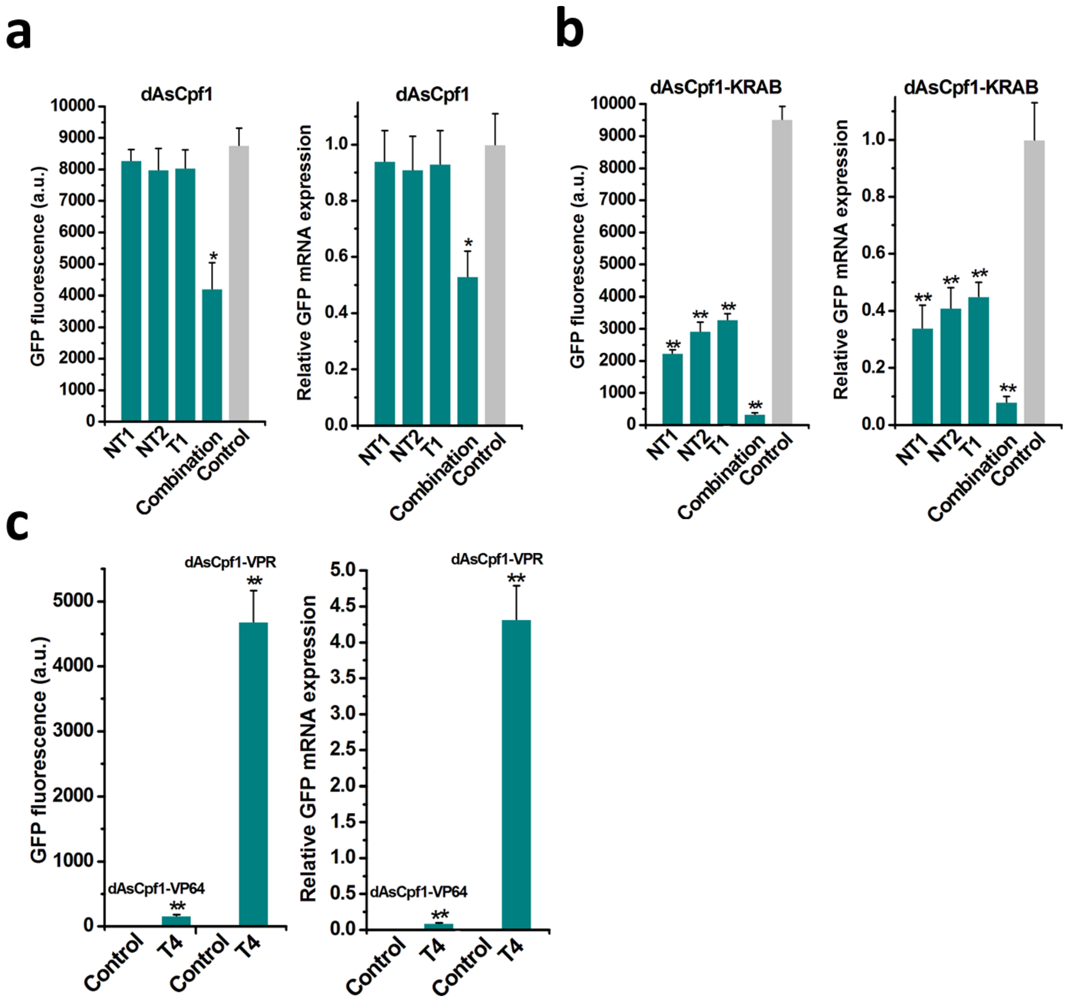

Supplementary Figure 3. Quantification of GFP signals in cells treated by the dAsCpf1-based transcription factors. Both FACS analysis and qRT-PCR have been performed for GFP activation and repression studies. Results are shown as mean ± standard deviation (S.D.) (n=5). *P < 0.05 (**P < 0.01) compared to non-target crRNA control by paired, one-sided t-test. a) GFP repression by the dAsCpf1 system. b) GFP repression by the dAsCpf1-KRAB system. c) GFP activation by the dAsCpf1-VP64 or by the dAsCpf1-VPR system.

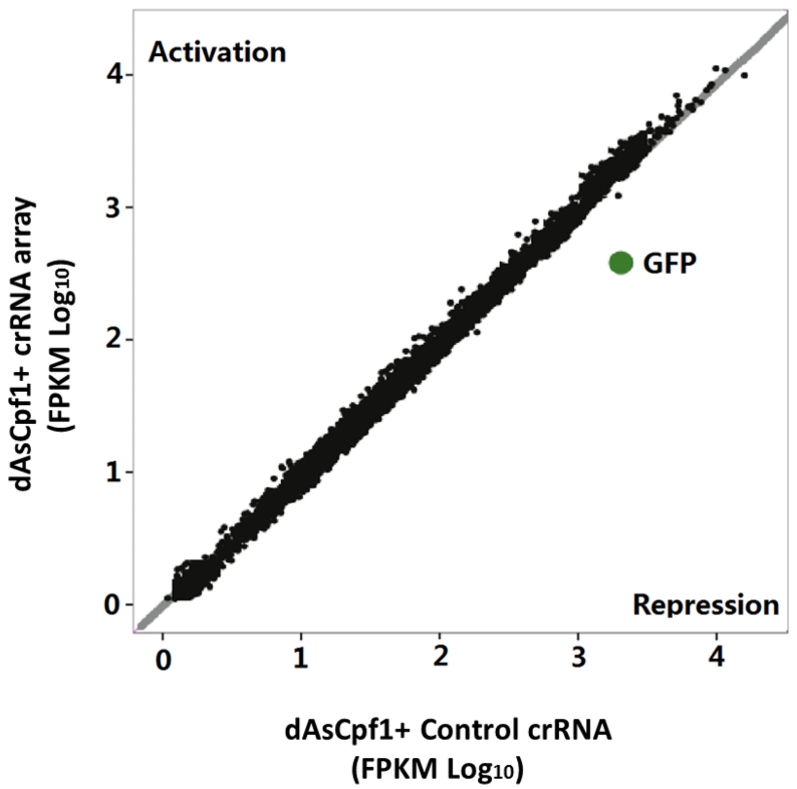

Supplementary Figure 4. Analysis of the specificity of dAsCpf1-mediated repression. HEK 293T cells expressing dAsCpf1 with the crRNA array or the control crRNA were analyzed. According to the FPKM values, only the transcription of the GFP was remarkably inhibited, demonstrating the high specificity of dAsCpf1-based transcription factors. GFP gene was highlighted. The data are representative of two independent biological replicates.

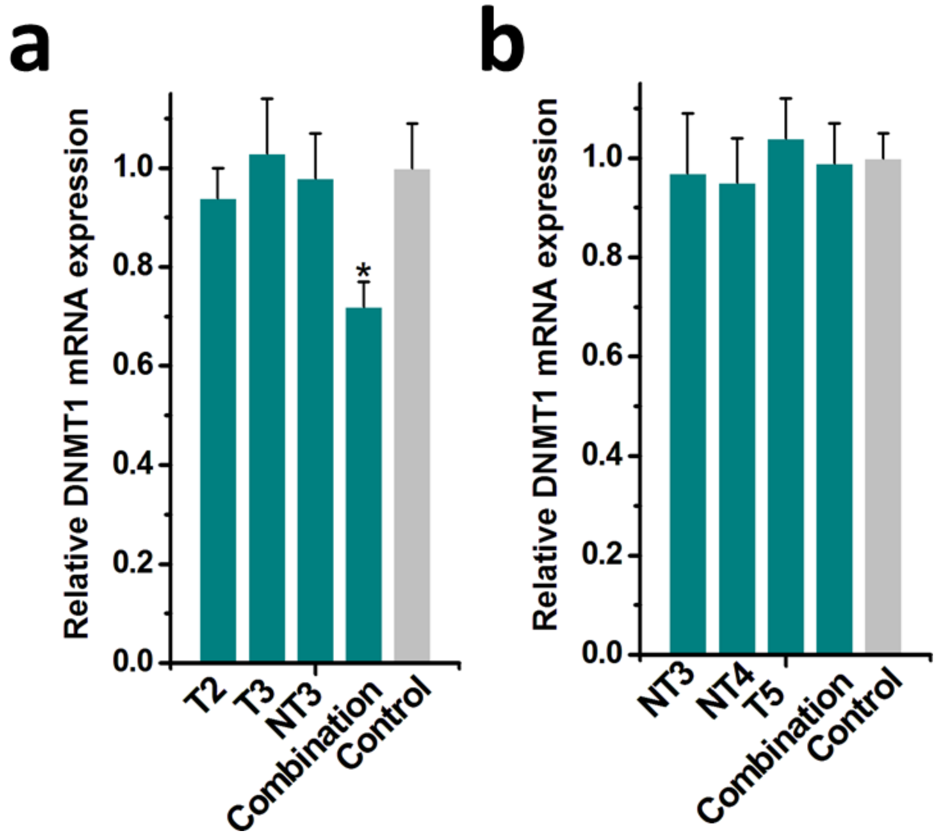

Supplementary Figure 5. dAsCpf1-mediated regulation of the DNMT1 expression in HEK293T cells. For DNMT1 we designed several crRNAs and a crRNA array expressing a combination of these crRNAs, and assayed transcriptional repression (a) or activation (b) by qRT-PCR. Reported data are mean ± SD from five independent experiments. *P < 0.05 compared to non-target crRNA control by paired, one-sided t-test.

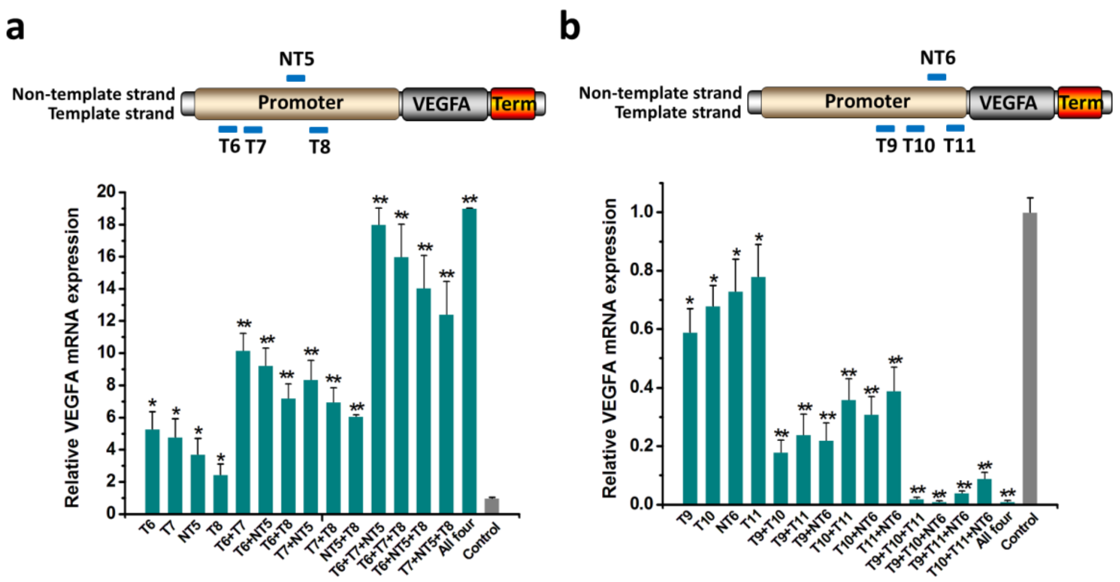

Supplementary Figure 6. dAsCpf1-based transcription factors-mediated regulation of the VEGFA expression in Hela cells. Based on the sequence of VEGFA promoter, we designed several crRNAs and tested their efficiencies on transcriptional activation and repression by qRT-PCR. Reported data are mean ± SD from five independent experiments. *P < 0.05 compared to non-target crRNA control by paired, one-sided t-test. **P < 0.01 compared to non-target crRNA control by paired, one-sided t-test. a) All four crRNAs induced significant increases in VEGFA transcript levels. Coexpression of dAsCpf1-VPR with subsets of two of these crRNAs induced synergistic activation of VEGFA expression. The crRNA array expressing three (T6+T7+NT5) or all of the four crRNAs led to the largest increases in VEGFA expression. b) All four crRNAs induced significant decreases in VEGFA transcript levels. Coexpression of dAsCpf1-KRAB with subsets of two of these crRNAs induced synergistic repression of VEGFA expression. The crRNA array expressing three (T9+T10+NT6) or all of the four crRNAs led to the largest decreases in VEGFA expression.

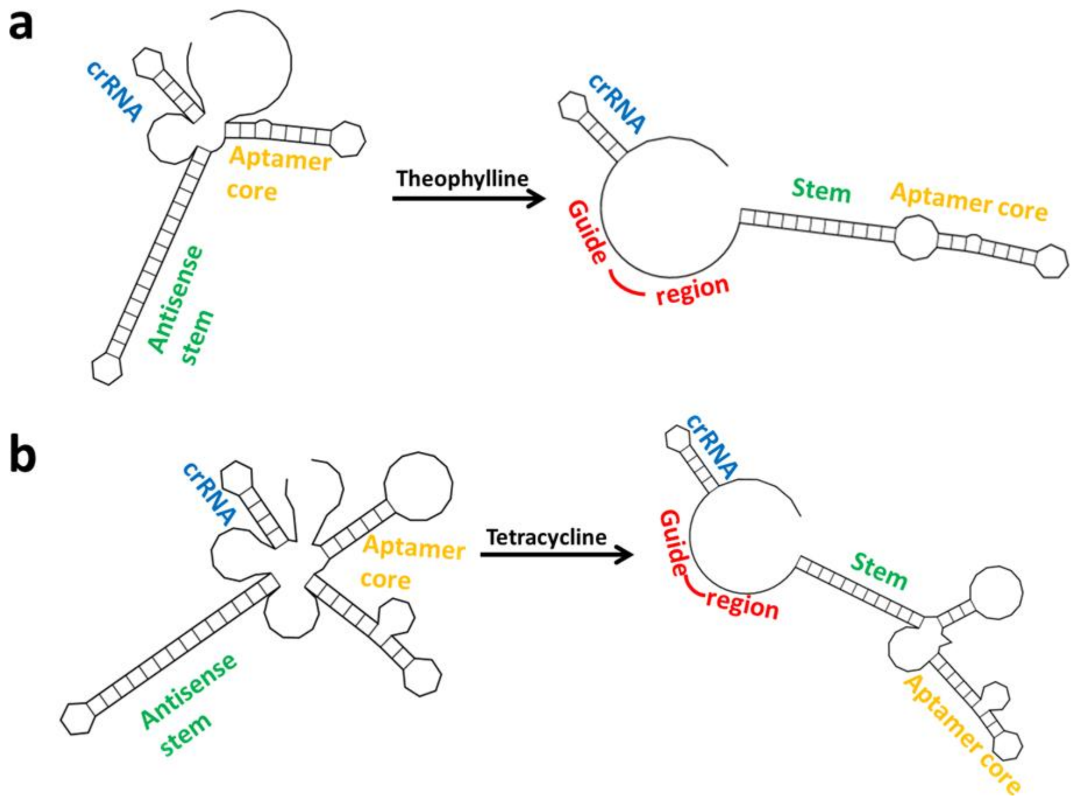

Supplementary Figure 7. The secondary structures of reprogrammed crRNAs as predicted by the RNAfold program. The crRNAs underwent ligand-catalyzed transformations to interact with the target gene. The guide region was exposed to the target DNA sequence only in the presence of the corresponding ligand. a) The modular structure of the crRNA used in theophylline-inducible constructs. b) The modular structure of the crRNA used in tetracycline-inducible constructs.

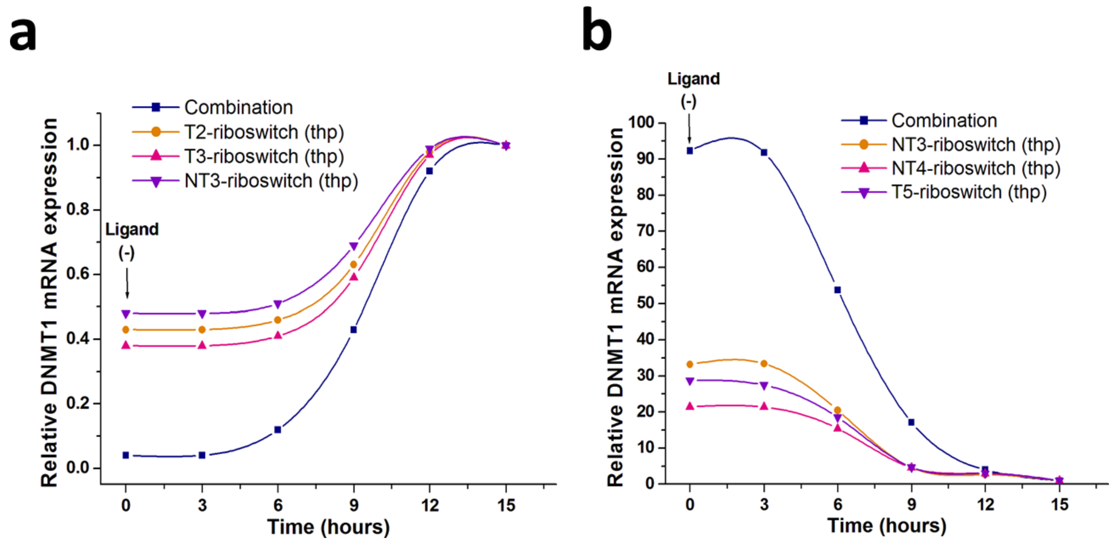

Extended Data Figure 8. The regulation of crRNA-riboswitch system is reversible. At Time = 0, theophylline was washed away from the growth media. The DNMT1 expression levels at various time points were measured by qRT-PCR. a) DNMT1 mRNA started to increase after 3 h and took about 15 h to rise to the same level as the original state. b) DNMT1 mRNA started to decrease after 3 h and took about 15 h to reduce to the same level as the original state.

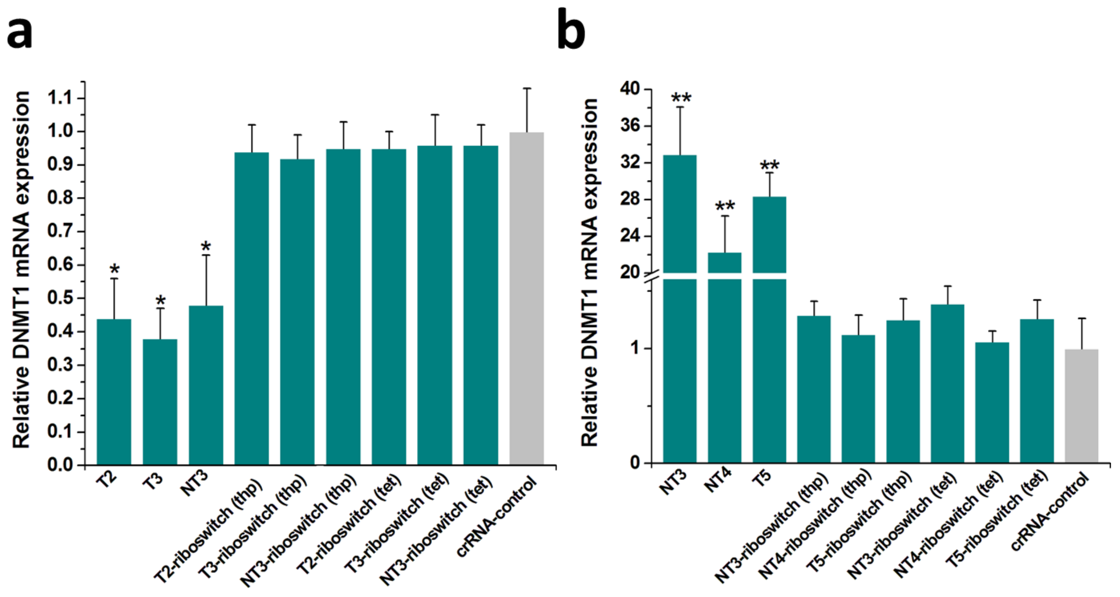

Supplementary Figure 9. The basal activity of crRNA-riboswitch system compared to unmodified dAsCpf1-system. dAsCpf1-KRAB was used for repression studies and dAsCpf1-VPR was used for activation studies. The DNMT1 expression level, related to the internal control (TBP), was measured by qRT-PCR. Reported data are mean ± SD from five independent experiments. *P < 0.05 compared to non-target crRNA control by paired, one-sided t-test. **P < 0.01 compared to non-target crRNA control by paired, one-sided t-test. a) There was no obvious reduction in DNMT1 expression when each crRNA-riboswitch construct was expressed without the presence of corresponding ligand. b) There was no obvious activation in DNMT1 expression when each crRNA-riboswitch construct was expressed without the presence of corresponding ligand.

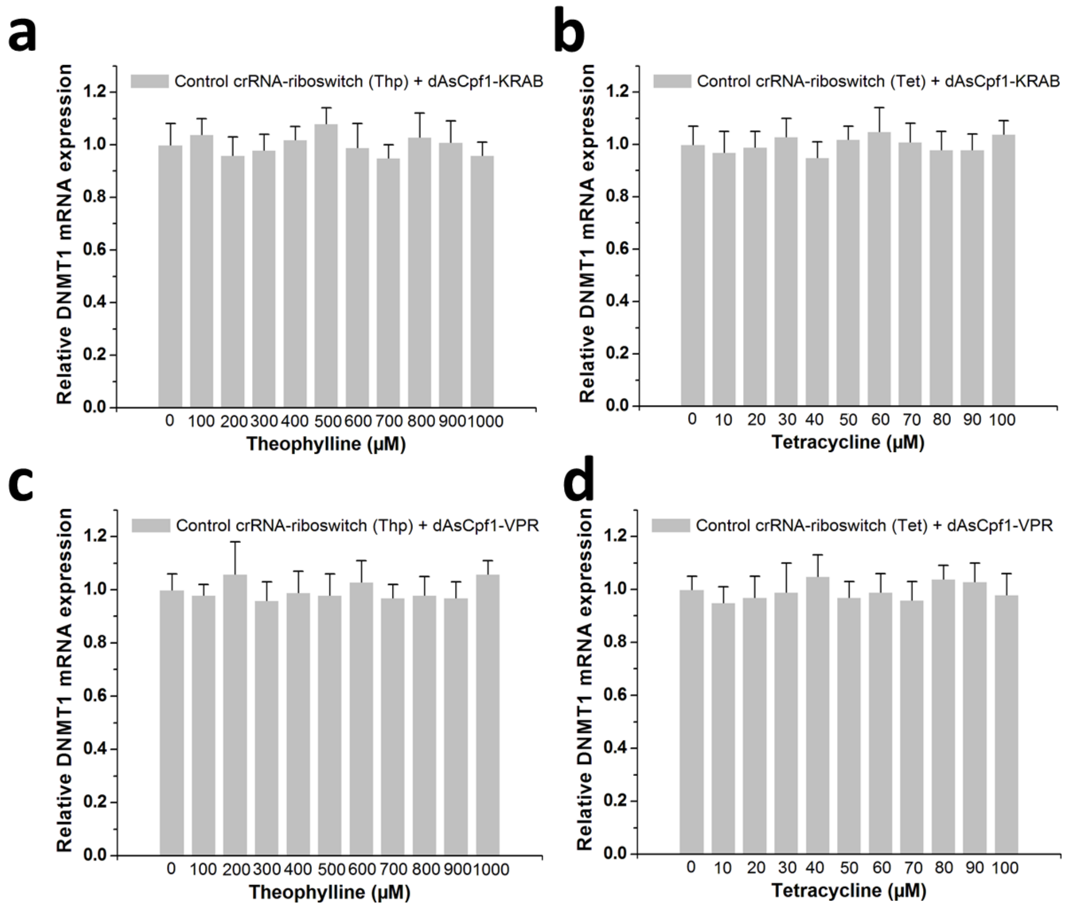

Supplementary Figure 10. The regulatory efficiencies of the control crRNA-riboswitches. The relative expression levels of DNMT1 mRNA were detected using real-time qPCR in HEK-293T cells that respond to theophylline or tetracycline across different concentrations. Reported data are mean ± SD from five independent experiments.

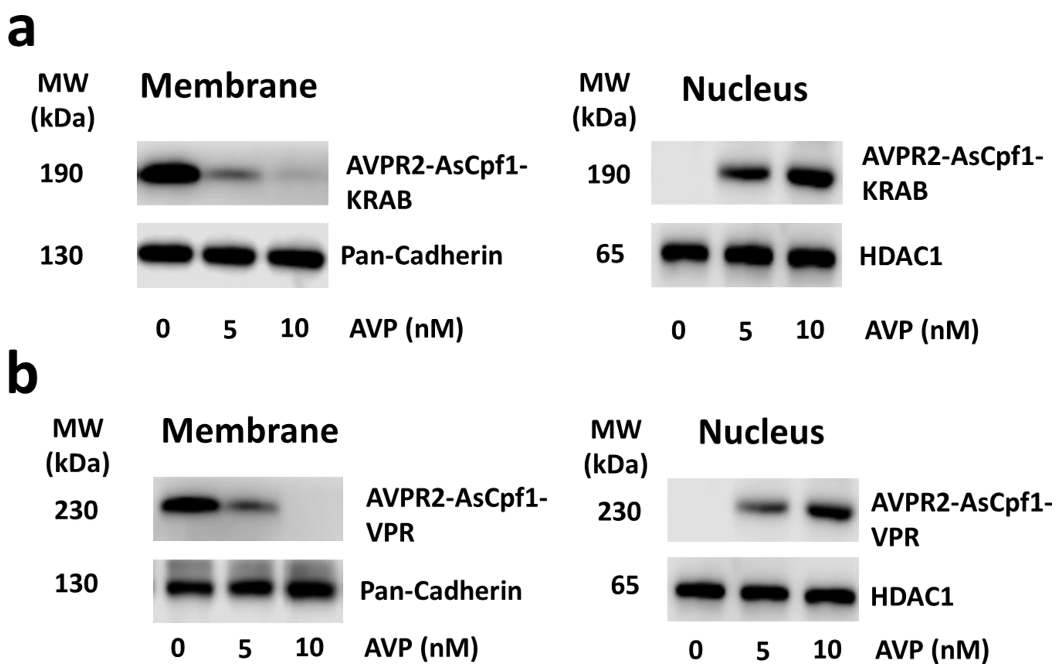

Supplementary Figure 11. Representative western blot analysis of AVPR2--dAsCpf1-KRAB or AVPR2- dAsCpf1-VPR in HEK293T cells. Proteins were extracted 24h post-transfection. dAsCpf1-fusion protein levels in membrane and nuclear proteins from HEK293T cells were detected by western blot. Two marker proteins were used as the internal controls (pan-cadherin for membrane and histone deacetylase 1 (HDAC1) for nucleus). Upon AVP ligand (5nM or 10nM) treatment, AVPR2-dAsCpf1-KRAB or AVPR2-dAsCpf1-VPR was translocated from the cell membrane receptor to the nucleus. In each experiment all samples were run in the same blot.

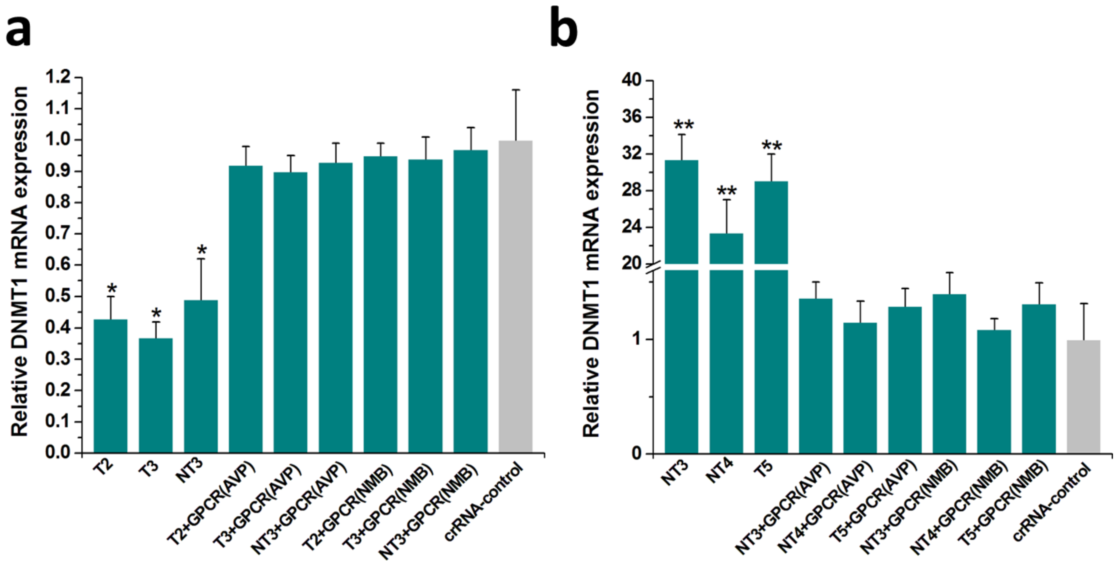

Supplementary Figure 12. The basal activity of dAsCpf1-GPCR system compared to unmodified dAsCpf1-system. dAsCpf1-KRAB was used for repression studies and dAsCpf1-VPR was used for activation studies. The DNMT1 expression level, related to the internal control (TBP), was measured by qRT-PCR. Reported data are mean ± SD from five independent experiments. *P < 0.05 compared to non-target crRNA control by paired, one-sided t-test. **P < 0.01 compared to non-target crRNA control by paired, one-sided t-test. a) There was no obvious reduction in DNMT1 expression when each dAsCpf1-GPCR construct was expressed without the presence of corresponding ligand. b) There was no obvious activation in DNMT1 expression when each dAsCpf1-GPCR construct was expressed without the presence of corresponding ligand.

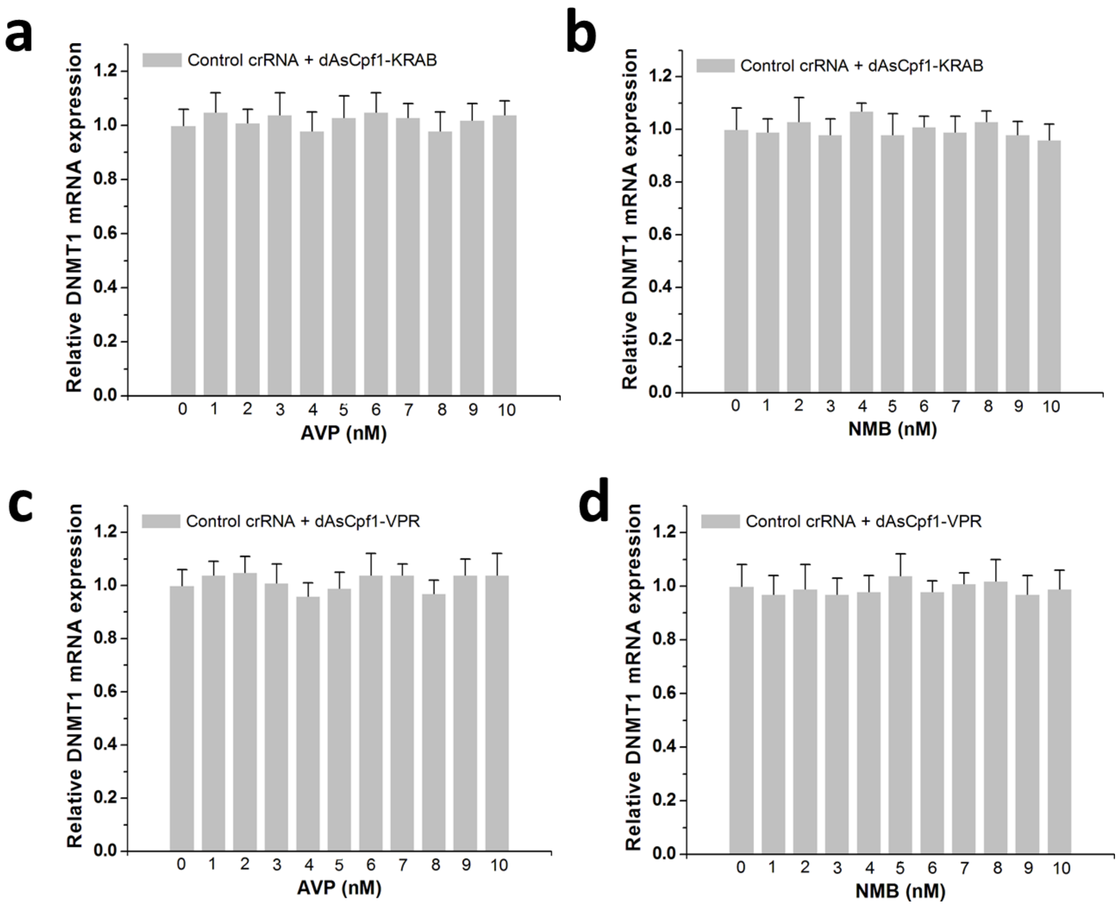

Supplementary Figure 13. The regulatory efficiencies of the control GPCR systems. The relative expression levels of DNMT1 mRNA were detected using real-time qPCR in HEK-293T cells that respond to AVP or NMB across different concentrations. Reported data are mean ± SD from five independent experiments.

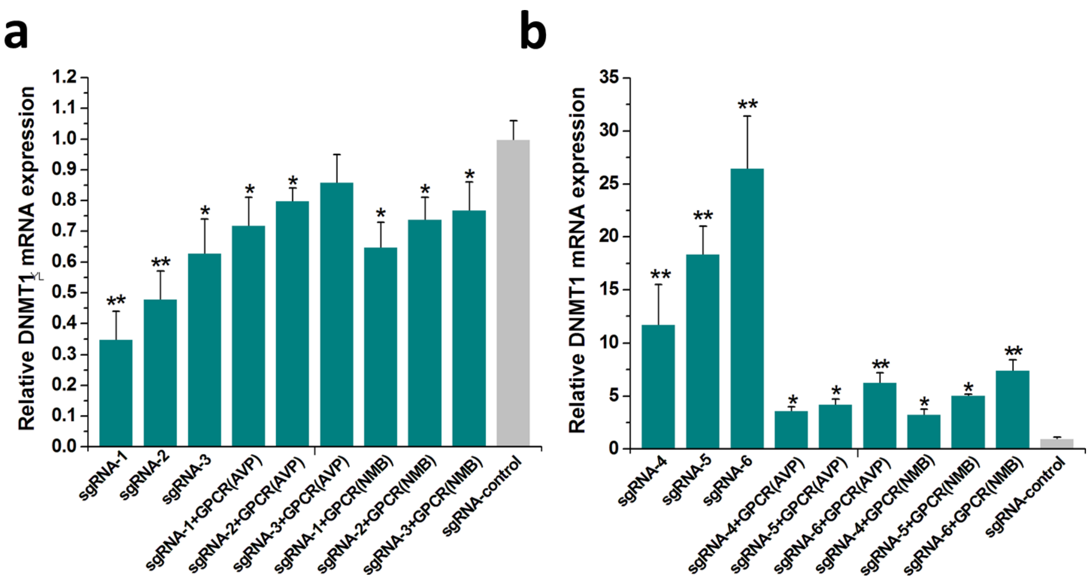

Supplementary Figure 14. The basal activity of dCas9-GPCR system compared to unmodified dCas9-system. dCs9-KRAB was used for repression studies and dCas9-VPR was used for activation studies. The DNMT1 expression level, related to the internal control (TBP), was measured by qRT-PCR. Reported data are mean ± SD from five independent experiments. *P < 0.05 compared to non-target crRNA control by paired, one-sided t-test. **P < 0.01 compared to non-target crRNA control by paired, one-sided t-test. a) There was modest reduction in DNMT1 expression when each dCas9-GPCR construct was expressed without the presence of corresponding ligand. b) There was modest activation in DNMT1 expression when each dCas9-GPCR construct was expressed without the presence of corresponding ligand.

Supplementary Table 1. cDNA sequences of the crRNAs used in this study. Each of these sequences may consist of a crRNA sequence, a crRNA guide region for DNA binding, a linker sequence, two aptamer stems and an aptamer core.

Names	Sequences
control crRNA	TAATTTCTACTCTTGTAGATACTCCTCGTTCACCGCCGTCGCG
NT1	TAATTTCTACTCTTGTAGATGGAGCGCGCCGCTCTGATTGGCT
NT2	TAATTTCTACTCTTGTAGATACGCAGCCACAGAAAAGAAACGA
NT3	TAATTTCTACTCTTGTAGATCTGGGAGGTGGGCACGGTGCCCG
NT4	TAATTTCTACTCTTGTAGATCTGGCTATACGACCTTAGGTGGG
NT5	TAATTTCTACTCTTGTAGATAAAAAAAAAAGGGGGGGGCGCAT
NT6	TAATTTCTACTCTTGTAGATAGAAAAAAGAAGAGGGATAAAAC
T1	TAATTTCTACTCTTGTAGATTGGTAATCGTGCGAGAGGGCGCA
T2	TAATTTCTACTCTTGTAGATCGCGCGAAAAGCCGGGGCGCCTG
T3	TAATTTCTACTCTTGTAGATGTACATCCCCTCCTCCCCCACGC
T4	TAATTTCTACTCTTGTAGATCTCCCTATCAGTGATAGAGAACG
T5	TAATTTCTACTCTTGTAGATCTCAAGGGCTCTCACAAACCCTT
T6	TAATTTCTACTCTTGTAGATCGGGGGCGGATGGGTAATTTTCA
T7	TAATTTCTACTCTTGTAGATAGGCTGTGAACCTTGGTGGGGGT
T8	TAATTTCTACTCTTGTAGATTTTAAAAGTCGGCTGGTAGCGGG
T9	TAATTTCTACTCTTGTAGATAAAGTCGGCTGGTAGCGGGGAGG
T10	TAATTTCTACTCTTGTAGATATATTCATTGATCCGGGTTTTAT
T11	TAATTTCTACTCTTGTAGATTTAAAACTGTATTGTTTCTCGTT

T2-riboswitch (thp)	TAATTTCTACTCTTGTAGATCGCGCGAAAAGCCGGGCGCCTG CCTCCAGGCGCCCGGCTTGATACCAGCATCGTCTTGATGCCCT TGGCAGCAAGCCGGGC
T3-riboswitch (thp)	TAATTTCTACTCTTGTAGATGTACATCCCCTCCTCCCCACGCCC TCGCGTGGGGAGGAGGGATACCAGCATCGTCTTGATGCCC TTGGCAGCCCTCCTCCCC
NT3-riboswitch (thp)	TAATTTCTACTCTTGTAGATCTGGGAGGTGGGCACGGTGCCCGC CTCCGGGCACCGTGCCCAGATACCAGCATCGTCTTGATGCCCTT GGCAGCTGGGCACGGT
NT4-riboswitch (thp)	TAATTTCTACTCTTGTAGATCTGGCTATACGACCTTAGGTGGGCC TCCCCACCTAAGGTCGTGATACCAGCATCGTCTTGATGCCCTTG GCAGCACGACCTTAG
NT5-riboswitch (thp)	TAATTTCTACTCTTGTAGATAAAAAAAAAAGGGGGGGGCGCAT CCTCATGCGCCCCCCCTTGATACCAGCATCGTCTTGATGCCCT TGGCAGCAAGGGGGGGG
control crRNA-riboswitch (thp)	TAATTTCTACTCTTGTAGATACTCCTCGTTCACCGCCGTCGCGCC TCCGCGACGGCGGTGAAGATACCAGCATCGTCTTGATGCCCTT GGCAGCTTCACCGCCG
T2-riboswitch (tet)	TAATTTCTACTCTTGTAGATCGCGCGAAAAGCCGGGCGCCTG CCTCCAGGCGCCCGGCTTAAAACATACCAGATCGCCACCCG CGCTTTAATCTGGAGAGGTGAAGAATACGACCACCTAAGCCGG GGC
T3-riboswitch (tet)	TAATTTCTACTCTTGTAGATGTACATCCCCTCCTCCCCACGCCC TCGCGTGGGGAGGAGGAAAACATACCAGATCGCCACCCGCG CTTTAATCTGGAGAGGTGAAGAATACGACCACCTCCTCCTCCC
NT3-riboswitch (tet)	TAATTTCTACTCTTGTAGATCTGGGAGGTGGGCACGGTGCCCGC CTCCGGGCACCGTGCCCAAAAACATACCAGATCGCCACCCGCG CTTTAATCTGGAGAGGTGAAGAATACGACCACCTTGGGCACGG T
NT4-riboswitch (tet)	TAATTTCTACTCTTGTAGATCTGGCTATACGACCTTAGGTGGGCC TCCCCACCTAAGGTCGTAAAACATACCAGATCGCCACCCGCGC TTTAATCTGGAGAGGTGAAGAATACGACCACCTACGACCTTAG
NT5-riboswitch (tet)	TAATTTCTACTCTTGTAGATAAAAAAAAAAGGGGGGGGCGCAT CCTCATGCGCCCCCCCTTAAAACATACCAGATCGCCACCCGCG CTTTAATCTGGAGAGGTGAAGAATACGACCACCTAAGGGGGGG G
control crRNA-riboswitch (tet)	TAATTTCTACTCTTGTAGATACTCCTCGTTCACCGCCGTCGCGCC TCCGCGACGGCGGTGAAAAAACATACCAGATCGCCACCCGCGC TTTAATCTGGAGAGGTGAAGAATACGACCACCTCTTCACCGCC G

Supplementary Table 2. Spacer Sequences for sgRNAs used in dCas9-GPCRs.

Names	Sequences
control sgRNA	GTACGTTCTCTATCACTGATA
sgRNA1	GACATCGTCGGGCAGCGAGA
sgRNA 2	GCGCGGAAACCGATGGGGAG
sgRNA 3	GGTTTCCGCGCGAAAAGCCG
sgRNA 4	GGGAACACGCATGCGCAAGG
sgRNA 5	GTGGGCTCGTCCAAGCTCCA
sgRNA 6	GCTGGCTATACGACCTTAGG

Supplementary Note 1. The cDNA sequence of dAsCpf1 used in this study.

> Human-codon optimized dAsCpf1 (D908A)

ATGACACAGTTCGAGGGCTTTACCAACCTGTATCAGGTGAGCAAGACACTGCGGTTTG
AGCTGATCCCACAGGGCAAGACCCTGAAGCACATCCAGGAGCAGGGCTTCATCGAGG
AGGACAAGGCCCGCAATGATCACTACAAGGAGCTGAAGCCCATCATCGATCGGATCTA
CAAGACCTATGCCGACCAGTGCCTGCAGCTGGTGCAGCTGGATTGGGAGAACCTGAG
CGCCGCCATCGACTCCTATAGAAAGGAGAAAACCGAGGAGACAAGGAACGCCCTGAT
CGAGGAGCAGGCCACATATCGCAATGCCATCCACGACTACTTCATCGGCCGGACAGAC
AACCTGACCGATGCCATCAATAAGAGACACGCCGAGATCTACAAGGGCCTGTTCAAGG
CCGAGCTGTTTAATGGCAAGGTGCTGAAGCAGCTGGGCACCGTGACCACAACCGAGC
ACGAGAACGCCCTGCTGCGGAGCTTCGACAAGTTTACAACCTACTTCTCCGGCTTTTAT
GAGAACAGGAAGAACGTGTTCAGCGCCGAGGATATCAGCACAGCCATCCCACACCGC
ATCGTGCAGGACAACTTCCCCAAGTTTAAGGAGAATTGTCACATCTTCACACGCCTGAT
CACCGCCGTGCCCAGCCTGCGGGAGCACTTTGAGAACGTGAAGAAGGCCATCGGCAT
CTTCGTGAGCACCTCCATCGAGGAGGTGTTTTCCTTCCCTTTTTATAACCAGCTGCTGA
CACAGACCCAGATCGACCTGTATAACCAGCTGCTGGGAGGAATCTCTCGGGAGGCAGG
CACCGAGAAGATCAAGGGCCTGAACGAGGTGCTGAATCTGGCCATCCAGAAGAATGA
TGAGACAGCCCACATCATCGCCTCCCTGCCACACAGATTCATCCCCCTGTTTAAGCAGA
TCCTGTCCGATAGGAACACCCTGTCTTTCATCCTGGAGGAGTTTAAGAGCGACGAGGA
AGTGATCCAGTCCTTCTGCAAGTACAAGACACTGCTGAGAAACGAGAACGTGCTGGA
GACAGCCGAGGCCCTGTTTAACGAGCTGAACAGCATCGACCTGACACACATCTTCATC
AGCCACAAGAAGCTGGAGACAATCAGCAGCGCCCTGTGCGACCACTGGGATACACTG
AGGAATGCCCTGTATGAGCGGAGAATCTCCGAGCTGACAGGCAAGATCACCAAGTCTG
CCAAGGAGAAGGTGCAGCGCAGCCTGAAGCACGAGGATATCAACCTGCAGGAGATCA
TCTCTGCCGCAGGCAAGGAGCTGAGCGAGGCCTTCAAGCAGAAAACCAGCGAGATCC
TGTCCCACGCACACGCCGCCCTGGATCAGCCACTGCCTACAACCCTGAAGAAGCAGG
AGGAGAAGGAGATCCTGAAGTCTCAGCTGGACAGCCTGCTGGGCCTGTACCACCTGC
TGGACTGGTTTGCCGTGGATGAGTCCAACGAGGTGGACCCCGAGTTCTCTGCCCGGCT
GACCGGCATCAAGCTGGAGATGGAGCCTTCTCTGAGCTTCTACAACAAGGCCAGAAAT
TATGCCACCAAGAAGCCCTACTCCGTGGAGAAGTTCAAGCTGAACTTTCAGATGCCTA
CACTGGCCTCTGGCTGGGACGTGAATAAGGAGAAGAACAATGGCGCCATCCTGTTTGT
GAAGAACGGCCTGTACTATCTGGGCATCATGCCAAAGCAGAAGGGCAGGTATAAGGCC
CTGAGCTTCGAGCCCACAGAGAAAACCAGCGAGGGCTTTGATAAGATGTACTATGACT
ACTTCCCTGATGCCGCCAAGATGATCCCAAAGTGCAGCACCCAGCTGAAGGCCGTGAC
AGCCCACTTTCAGACCCACACAACCCCCATCCTGCTGTCCAACAATTTCATCGAGCCTC
TGGAGATCACAAAGGAGATCTACGACCTGAACAATCCTGAGAAGGAGCCAAAGAAGT
TTCAGACAGCCTACGCCAAGAAAACCGGCGACCAGAAGGGCTACAGAGAGGCCCTGT
GCAAGTGGATCGACTTCACAAGGGATTTTCTGTCCAAGTATACCAAGACAACCTCTATC
GATCTGTCTAGCCTGCGGCCATCCTCTCAGTATAAGGACCTGGGCGAGTACTATGCCGA
GCTGAATCCCCTGCTGTACCACATCAGCTTCCAGAGAATCGCCGAGAAGGAGATCATG
GATGCCGTGGAGACAGGCAAGCTGTACCTGTTCCAGATCTATAACAAGGACTTTGCCA
AGGGCCACCACGGCAAGCCTAATCTGCACACACTGTATTGGACCGGCCTGTTTTCTCC

AGAGAACCTGGCCAAGACAAGCATCAAGCTGAATGGCCAGGCCGAGCTGTTCTACCG
CCCTAAGTCCAGGATGAAGAGGATGGCACACCGGCTGGGAGAGAAGATGCTGAACAA
GAAGCTGAAGGATCAGAAAACCCCAATCCCCGACACCCTGTACCAGGAGCTGTACGA
CTATGTGAATCACAGACTGTCCCACGACCTGTCTGATGAGGCCAGGGCCCTGCTGCCC
AACGTGATCACCAAGGAGGTGTCTCACGAGATCATCAAGGATAGGCGCTTTACCAGCG
ACAAGTTCTTTTTCCACGTGCCTATCACACTGAACTATCAGGCCGCCAATTCCCCATCT
AAGTTCAACCAGAGGGTGAATGCCTACCTGAAGGAGCACCCCGAGACACCTATCATCG
GCATCGCCCGGGGCGAGAGAAACCTGATCTATATCACAGTGATCGACTCCACCGGCAA
GATCCTGGAGCAGCGGAGCCTGAACACCATCCAGCAGTTTGATTACCAGAAGAAGCTG
GACAACAGGGAGAAGGAGAGGGTGGCAGCAAGGCAGGCCTGGTCTGTGGTGGGCAC
AATCAAGGATCTGAAGCAGGGCTATCTGAGCCAGGTCATCCACGAGATCGTGGACCTG
ATGATCCACTACCAGGCCGTGGTGGTGCTGGAGAACCTGAATTTCGGCTTTAAGAGCA
AGAGGACCGGCATCGCCGAGAAGGCCGTGTACCAGCAGTTCGAGAAGATGCTGATCG
ATAAGCTGAATTGCCTGGTGCTGAAGGACTATCCAGCAGAGAAAGTGGGAGGCGTGCT
GAACCCATACCAGCTGACAGACCAGTTCACCTCCTTTGCCAAGATGGGCACCCAGTCT
GGCTTCCTGTTTTACGTGCCTGCCCCATATACATCTAAGATCGATCCCCTGACCGGCTTC
GTGGACCCCTTCGTGTGGAAAACCATCAAGAATCACGAGAGCCGCAAGCACTTCCTG
GAGGGCTTCGACTTTCTGCACTACGACGTGAAAACCGGCGACTTCATCCTGCACTTTA
AGATGAACAGAAATCTGTCCTTCCAGAGGGGCCTGCCCGGCTTTATGCCTGCATGGGAT
ATCGTGTTCGAGAAGAACGAGACACAGTTTGACGCCAAGGGCACCCCTTTCATCGCCG
GCAAGAGAATCGTGCCAGTGATCGAGAATCACAGATTCACCGGCAGATACCGGGACCT
GTATCCTGCCAACGAGCTGATCGCCCTGCTGGAGGAGAAGGGCATCGTGTTCAGGGAT
GGCTCCAACATCCTGCCAAAGCTGCTGGAGAATGACGATTCTCACGCCATCGACACCA
TGGTGGCCCTGATCCGCAGCGTGCTGCAGATGCGGAACTCCAATGCCGCCACAGGCGA
GGACTATATCAACAGCCCCGTGCGCGATCTGAATGGCGTGTGCTTCGACTCCCGGTTTC
AGAACCCAGAGTGGCCCATGGACGCCGATGCCAATGGCGCCTACCACATCGCCCTGAA
GGGCCAGCTGCTGCTGAATCACCTGAAGGAGAGCAAGGATCTGAAGCTGCAGAACGG
CATCTCCAATCAGGACTGGCTGGCCTACATCCAGGAGCTGCGCAAC
